# Multimodal Artificial Synapses for Neuromorphic Application

**DOI:** 10.34133/research.0427

**Published:** 2024-08-19

**Authors:** Runze Li, Zengji Yue, Haitao Luan, Yibo Dong, Xi Chen, Min Gu

**Affiliations:** ^1^ School of Artificial Intelligence Science and Technology, University of Shanghai for Science and Technology, Shanghai 200093, China.; ^2^ Institute of Photonic Chips, University of Shanghai for Science and Technology, Shanghai 200093, China.; ^3^ Zhangjiang Laboratory, Pudong, Shanghai 201210, China.

## Abstract

The rapid development of neuromorphic computing has led to widespread investigation of artificial synapses. These synapses can perform parallel in-memory computing functions while transmitting signals, enabling low-energy and fast artificial intelligence. Robots are the most ideal endpoint for the application of artificial intelligence. In the human nervous system, there are different types of synapses for sensory input, allowing for signal preprocessing at the receiving end. Therefore, the development of anthropomorphic intelligent robots requires not only an artificial intelligence system as the brain but also the combination of multimodal artificial synapses for multisensory sensing, including visual, tactile, olfactory, auditory, and taste. This article reviews the working mechanisms of artificial synapses with different stimulation and response modalities, and presents their use in various neuromorphic tasks. We aim to provide researchers in this frontier field with a comprehensive understanding of multimodal artificial synapses.

## Introduction

**Fig. 1.**
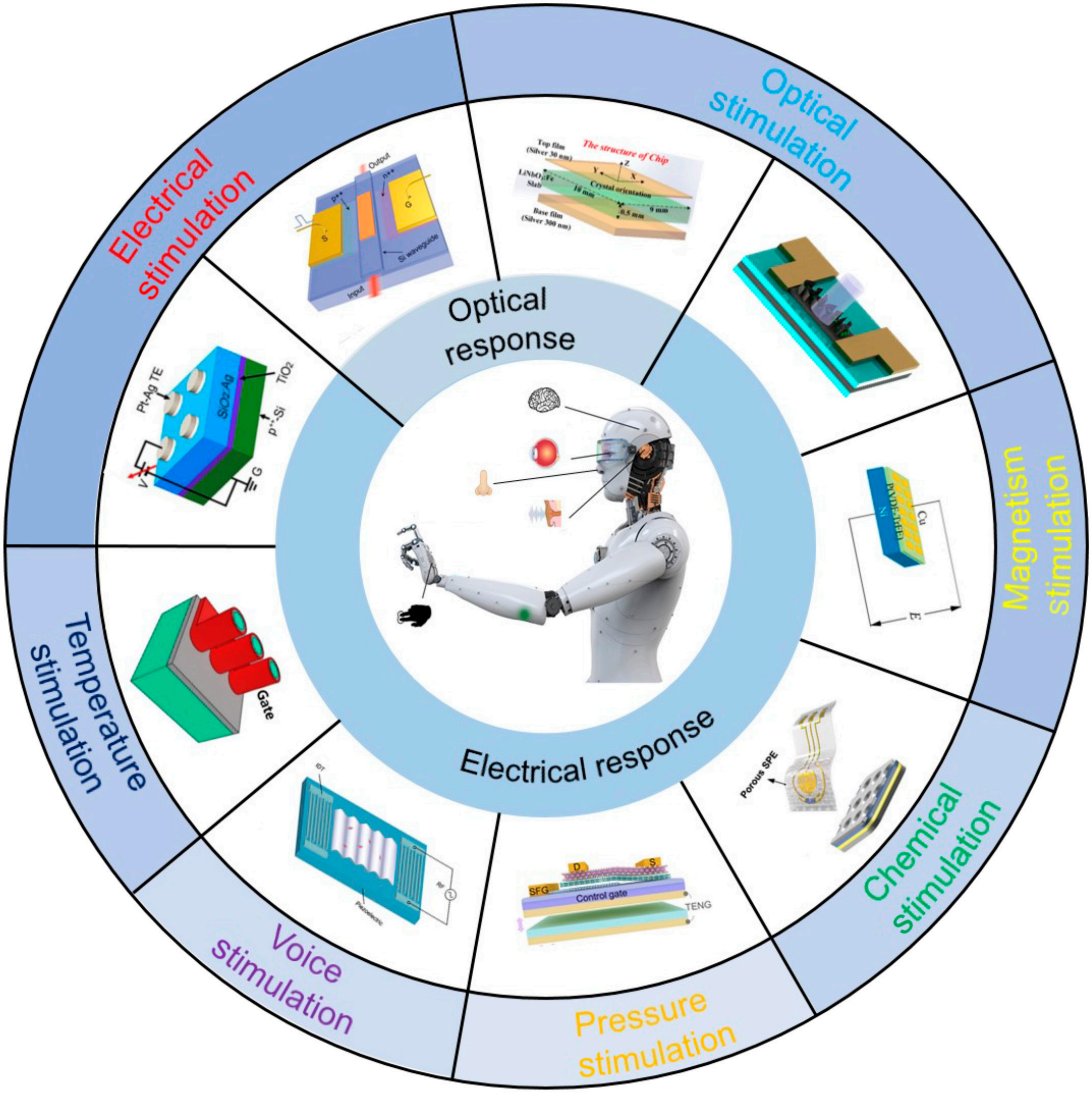
Classification of multimodal artificial synapses.

With the development of artificial intelligence (AI), neuromorphic systems capable of mimicking and performing intelligence functions have been extensively investigated [1–3]. AI has driven the evolution of a wide range of mechanical systems, especially robots. Equipped with AI, robots can be endowed with a certain level of intelligence and thus be capable of executing more complex tasks [4,5]. In order to truly replace humans in various jobs, intelligence robots require not only an AI system as the brain but also smarter sensory systems for visual, tactile, olfactory, auditory, and taste. Multimodal artificial synapse devices can serve as this role to achieve faster, lower-energy, and more anthropomorphic intelligent robots [6]. As shown in the center of Fig. 1, external information can be perceived through multiple types of artificial synapses, whereas cross-modal integrations occur in higher cortical areas.

Artificial synapses are devices that mimic the function of biological synapses. In human nervous system, different types of synapses can form excitatory postsynaptic currents (EPSCs) or inhibitory postsynaptic currents (IPSCs) by stimulation from different modal signals [7]. The strength of the connection between presynaptic neurons and postsynaptic neurons caused by neurotransmitters is defined as synaptic weight. Synaptic plasticity, which refers to the changes in synaptic weight caused by external stimulus signals, is considered the foundation of brain memory and learning [8]. For artificial synapses, the basic function is synaptic plasticity. The weights of them can be modulated by external multimodal stimuli, including electrical, optical, acoustic, temperature, and gas signals. Further, by compactly arranging and parallelly connecting artificial synapses in neural networks, large-scale brain-like computing can be achieved with in-memory architecture and spiking configuration. Compared with conventional computer systems, the neural networks composed of artificial synapses demonstrate stronger adaptability, faster computing speed, and lower energy consumption.

Here, we review the latest advancements in multimodal artificial synapses from their mechanism to neuromorphic application. The structures and overviews of these artificial synapses are categorized according to the different types of stimulus and response signals (Fig. 1). It is clear that the response signal of most artificial synapses is electrical. This aligns with the characteristics of the human nervous system, where all external information is eventually converted into bioelectrical signals for processing. In addition, artificial synapses with light response have also attracted widespread attention in recent years. It features low power consumption and high speed, but has lower integration and programmability compared with electrical counterparts [9]. This review is structured as follows. In the first part, we summarize and generalize the mechanisms of the different types of synapses and elaborate on their characteristics. In the second part, we present the typical neuromorphic applications based on artificial synapses. Finally, we provide an outlook on the potential challenges and opportunities that may arise during the development of artificial synapses.

## Categories of Artificial Synapses

### Electrically stimulated electrical-response artificial synapses

Electrical stimulated synapses have a relatively long research history and high maturity. We call them electrical artificial synapses (EASs). The synaptic plasticity of EAS is manifested in that their resistance can be programmed with a memory effect by electrical stimulation, which means that the resistance will continue to remain for a certain period of time after the stimulus disappears. This can be achieved by various physical mechanisms, such as capacitance, resistance, and magnetoresistance. Depending on the electrode structure, artificial synapses based on electrical signal stimulation can be divided into 2 categories: 2-terminal and 3-terminal. Here, we focus on 2-terminal artificial synapses, namely, memristors.

Memristors are the most widely studied electrical synaptic devices, which represents the relationship between charge and magnetic flux. The basic effect of memristor is that the resistance changes with the amount of current passing through it. Even if the current stops, its resistance can remain. The multistate resistance changes and nonvolatile properties of memristors closely resemble synaptic functions in the biological nervous system. As 2-terminal devices, they can be easily integrated in large scale through the cross-bar circuit structure, thus enabling in-memory process vector input signals in parallel.

#### Ionic migration

**Fig. 2.**
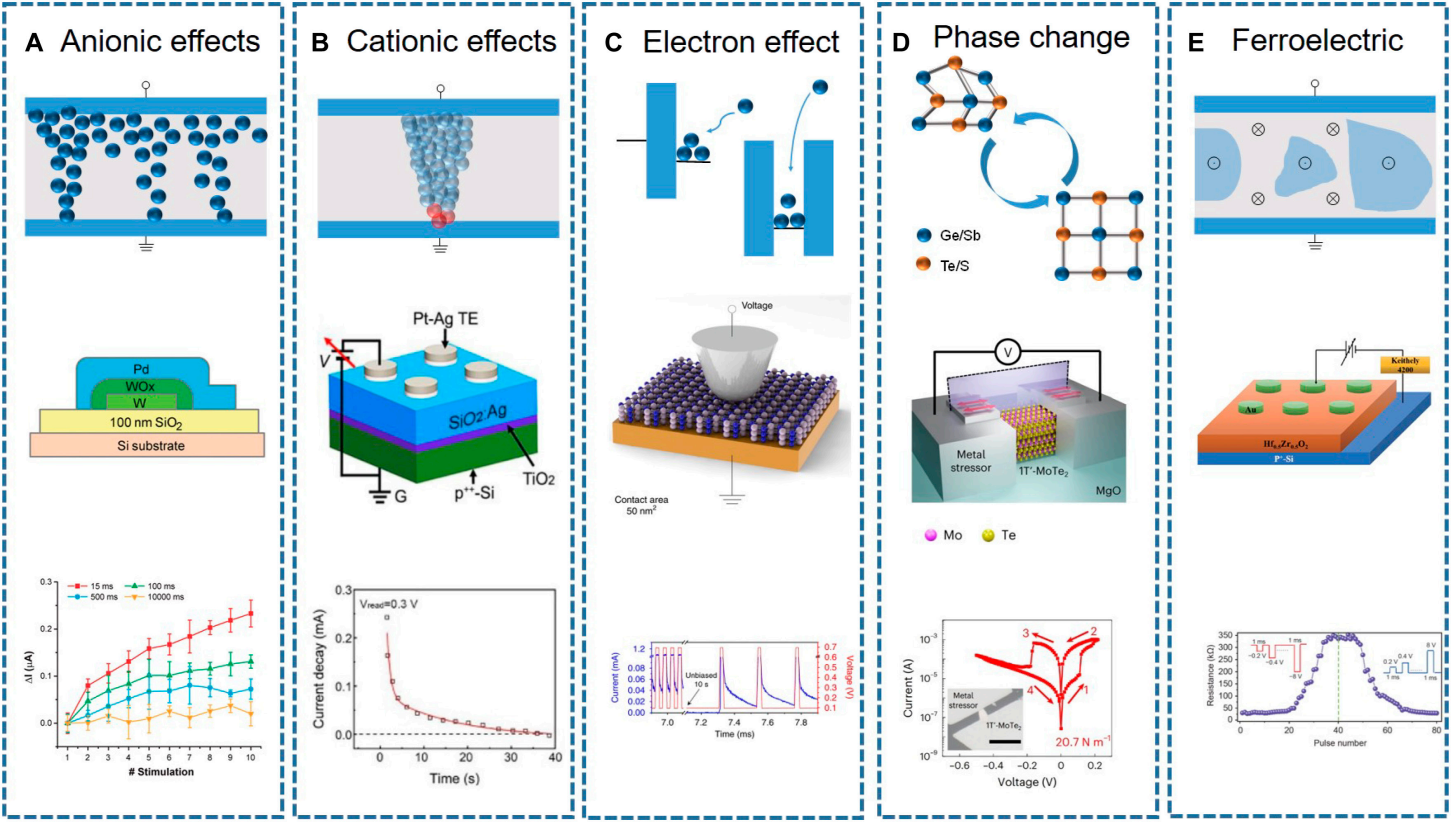
Mechanisms of synaptic plasticity in electrically stimulated electrical-response artificial synapses. (A) Anionic effects. (B) Cationic effects. (C) Electron effect. (D) Electro-induced phase transition. (E) Ferroelectric effects. Reprinted permission from [14,17,18,20,30], respectively.

Ionic effect includes anionic effects (e.g., O^2−^, -COO^−^, and I^−^) and cationic effects (e.g., Ag^+^, Cu^2+^, and other reactive metal ions). The dielectric layer of anionic effect memristors consists of oxide insulator materials, including transition metal oxides, perovskite-type complex oxides, and wide bandgap insulators [10–13]. Chang et al. [14] reported a nanoscale memristive device based on a thin film of WO*_X_*. The schematic structure of the device is shown in Fig. 2A. Oxygen vacancies (VOs) conductive channel is formed by the migration of oxygen ions in the dielectric layer of oxide insulator under the action of an electric field. When a positive bias voltage is applied to the electrode (anode), it induces the migration of VO toward the relative bottom electrode with the increase of the conductivity of the channel. On the contrary, when a negative bias voltage is applied, the ions return to the active layer within the interfacial oxide layer with the disappearance of the conductive channels. The cationic effect mainly refers to the physical migration and electrochemical reactions of active metal ions [15]. Metal ions generated by electrochemical reactions are reduced and form metal conductive filaments (MCFs) that connect both ends of the electrodes. The formation/dissolution process of the conductive metal filaments corresponds to the switching between low resistance state (LRS) and high resistance state (HRS) [16]. Devices with high cation migration rate electrolytes experience an excess of ionic current, resulting in the growth of tapered conductive filaments from the active electrode toward the inert electrode. In nonconventional electrolyte devices (such as SiO_2_ and other oxides), the conductive filaments start to grow from the inert electrode, but during the growth process, a limited number of cations diffuse into the filaments, resulting in branching growth. It is worth noting that the dissolution of the conductive filaments occurs near the interface of the dielectric layer and the inert electrode. Li et al. [17] reported a dual-terminal memristor-enhanced Pt–Ag/TiO_2_:Ag/p^++^-Si memristor by inserting a thin layer of TiO_2_ into the resistive switching layer. The schematic of the formation process of MCF is shown in Fig. 2B. When a positive bias is applied to the Ag electrode, the oxidized Ag^+^ ions migrate to the inert TiO_2_ layer and are reduced back to Ag atoms. Therefore, the device is in LRS. When a negative voltage is applied, the conductive Ag wire breaks and the resistance state of the device returns to the HRS. After removing the electrical stimulation, the current gradually decreases and returns to the initial state in approximately 40 s.

#### Electron effect

The electron effect mechanism is fundamentally different from the cation/anion migration mechanism. The cation/anion migration mechanism is based on the migration of ions, involving chemical redox reactions and physical thermal effects during the process. In contrast, the electron effect generally involves only electron transfer. The transition between HRS and LRS is achieved by trapping carriers through traps, resulting in the changes of carrier concentration in the channel. Shi et al. [18] used the chemical vapor deposition (CVD) to grow multilayer hexagonal boron nitride (h-BN) as the resistive switching dielectric to fabricate high-performance bidirectional EAS. Under the influence of an electric field, h-BN generates boron vacancies that can be filled by metal ions from adjacent electrodes. The power consumption for the high-to-low resistance state transition of the device can be as low as 600 pW, and the switching time is less than 10 ns. Figure 2C illustrates the EPSC behavior and the relaxation process during the high-to-low resistance state transition.

#### Phase transition

Phase change materials (PCMs) can undergo phase change at a certain temperature. After the phase change, the lattice structure changes, which leads to changes in the electrical, optical, and/or other properties of the material (Fig. 2D). At the same time, by regulating the temperature or using other methods, the structure of the PCM can return to its initial state, thereby achieving switching between different memory states. PCMs are widely investigated in artificial synapses not only in electrically stimulated synapses but also in other types of synapses. Currently, commonly used PCMs include bulk materials, such as GST (Ge_2_Sb_2_Te_5_), GeSb, vanadium, and Sb_2_Te, as well as some 2-dimensional (2D) materials, like MoTe_2_ [19–21]. Taking GST as an example, it can switch between the crystalline and amorphous states, thus realizing the 2 resistance states of LRS and HRS [22]. There are many factors that influence the phase transitions, among which the electro-induced phase transition mechanism [23–25] and the thermo-induced phase transition mechanism [26,27] have received considerable attention. MoTe_2_ can switch between semiconducting (2H) and semimetallic (1T′) phases. Recently, Hou et al. [20] reported a MoTe_2_-based phase change memristor with an ultralow phase switching voltage (Fig. 2D). This is achieved by a strain-engineering technique, the contact metal applies strain to MoTe_2_, bringing the active region closer to the phase switching point. Therefore, the device can achieve phase switching with a relative low energy consumption. Their device exhibits excellent performance with a switching voltage of 90 mV and a switching energy of 150 aJ.

#### Ferroelectric or ferromagnet effect

Memristive models based on ferroelectric or ferromagnet effects have also been gradually developed and applied in the field of neuromorphic computing. Their memristive behavior is typically associated with the hysteresis properties exhibited by spontaneous polarization or spontaneous magnetization under electric field stimulation or magnetic field stimulation [28,29]. Ferroelectric tunneling is the primary underlying mechanism of these synapse. Yu et al. [30] proposed an Au/HZO/p+-Si ferroelectric memristor, which can be modulated to multiple resistance states by changing the polarization state of the ferroelectric material through applied pulse voltages. The device structure is schematically shown in Fig. 2E, in which p+-Si is used as the bottom electrode, HZO film is used as the tunneling layer, and Au is used as the upper electrode. The curve shows the resistance changes of the device under a series of positive and negative voltage pulses.

To summarize EAS, memristors, due to their conductivity that can be modulated with a memory characteristic, are widely used as EAS. In addition, memristors have the advantages of simple structure and nanoscale dimensions, making them well suited for high-density integration, which holds important importance for the on-chip realization of brain-inspired computing. Besides, EAS can realize fast switching speed (in the nanosecond range) and low switching energy consumption (approximately 1 pJ), allowing them to more effectively update weights. Besides, the nonlinear computational capability of memristor arrays has enabled them to be used in various AI tasks such as recognition [31] and classification [32,33].

### Optically stimulated electrical-response artificial synapses

Recently, researches on optically stimulated electrical-response artificial synapses have gained considerable attention. Artificial synapses, whose conductivity can be modulated by optical signals, are called optoelectronic artificial synapses (OEASs).

Using light as stimulation, OEASs offer the advantage of fast response speed, low energy consumption, and low cross talk [34]. The OEAS with unidirectional conduction can solve the sneak path issues that appeared in electrical stimulated synapses [35]. Besides, OEASs offer a noncontact input method, which is crucial for the further development of optical wireless communication [36]. More importantly, OEAS can emulate the retinal neurons in the human eye. Since about 80% information of the outside world is conveyed to the human brain through visual perception, the use of OEAS can seamlessly translate optical stimuli into electrical signals [37], enabling efficient real-time image preprocessing.

The synaptic plasticity of OEAS is manifested in that its resistance can be modulated by light stimulation, which is reflected in the photocurrent. To achieve the memory effect of photocurrent, it is necessary to extend the lifetime of photogenerated carriers. According to different mechanisms, OEAS can be categorized into different types.

#### VO ionization

The inherent persistent photoconductivity characteristics (PPCs) of oxide semiconductor materials are important for their applications in optoelectronics [38]. PPC effect is closely related to nonvolatile photo-conductance and is commonly observed in various oxide semiconductors. In general, there are nonconductive α-type defects and conductive β-type defects in n-type semiconductors, in which α-type defects generate defect localized states within the bandgap. Conductive β-type defects can induce electrons to the conduction band minimum, which occupy the perturbed host states below the conduction band minimum for relaxation processes [39]. However, certain unique defects can exhibit both α-type and β-type behaviors simultaneously, in which the nonconducting ground state is transformed into a metastable conducting state by optical stimulation [40]. VOs in oxide semiconductors exhibit insulating α-type behavior in neutral ground state (VO^0^) but display conductive β-type characteristics in their metastable ionized states (VO^1+^ or VO^2+^) [41]. The change from stable conducting state to metastable ionized state in VO can be caused by light illumination when the photon energy exceeds the bandgap of the oxide semiconductors. Thereafter, when the light stimulus disappears, the electrons in the metastable ionized state will return to the stable state. This process is low because the electrons in the metastable ionized state need to circumvent a barrier (*E*_a_), resulting in the current memory effect.

**Fig. 3.**
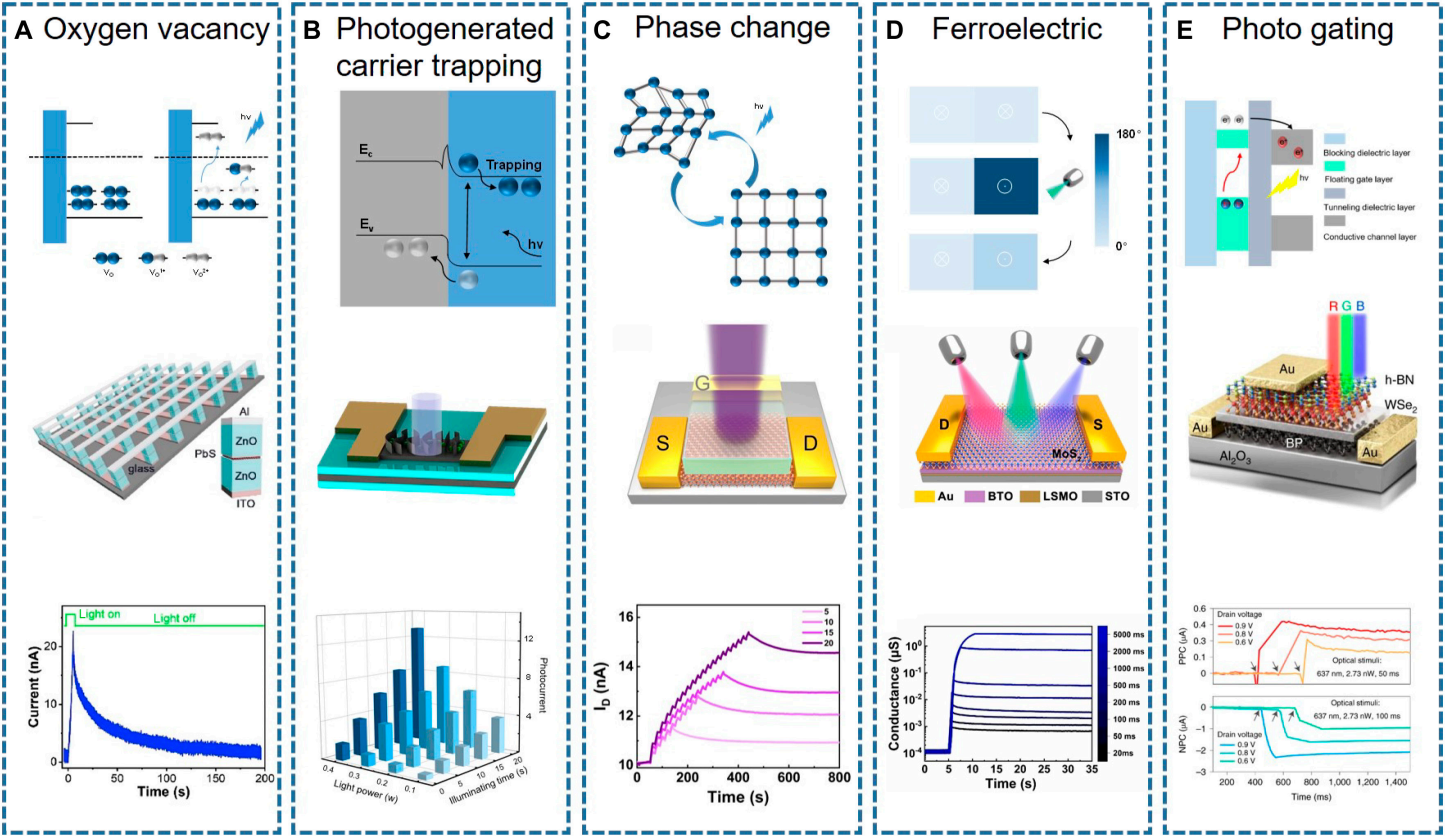
Mechanisms of synaptic plasticity in optically stimulated electrical-response artificial synapses. (A) Oxygen vacancy ionization. (B) Capture and release of photo-generated charge carriers. (C) Photo-induced phase transition. (D) Interaction between light and ferroelectric materials. (E) Photo-gating effect realized by building a floating gate. Reprinted permission from [42,45,59,77,79], respectively.

Li et al. [42] developed an artificial synaptic device based on a ZnO/PbS heterojunction with the schematic structure shown in Fig. 3A. In this device, ultraviolet (UV) light can induce EPSCs, while infrared light can induce IPSCs. Besides, other synaptic behaviors, like long-term plasticity (LTP), short-term plasticity (STP), paired-pulse facilitation (PPF), and paired-pulse depression (PPD), are also demonstrated [18]. Under UV light illumination, VOs^0^ are ionized to form VOs^1+^ or VOs^2+^, which are gradually increased by increasing the number of pulses or prolonging the light exposure time, leading to the generation of EPSCs. In contrast, infrared light can suppress the generation of ionized VOs, therefore resulting in inhibitory effects. After stimulation with light pulses of UV light, the EPSCs can exhibit a relaxation time over 150 s.

#### Photogenerated carrier capture and/or release

By designing the device structure or material to capture photogenerated electrons or holes, the recombination of photogenerated carriers can be effectively slowed down, thereby extending their lifetime. At present, the commonly used methods include introducing defects or building heterojunction.

1. Defect-induced photogenerated carrier capture

Defects can form traps capable of capturing photogenerated electrons or holes, thereby slowing down the recombination of photogenerated carriers. There are many methods that can introduce defects in materials including using lattice defects of materials, using surface dangling bonds of nanomaterials, and using interface defects [43–45].

For using lattice defects of materials, Li et al. [45] developed such OEAS device based on CsPbBr_3_ quantum dots (QDs) and graphene nanowalls. The graphene nanowalls were grown by plasma-assisted CVD and has a certain amount of defects, which naturally have a photocurrent memory effect [46]. They further extended the lifetime of photogenerated carriers by introducing a heterojunction by coating CsPbBr_3_ QDs on graphene nanowalls. Figure 3B illustrates the device structure and the photocurrent under dual-pulsed laser stimulation. For using surface dangling bonds, Tan et al. [44] reported a 3-terminal OEAS using Si nanocrystals (Si NCs). The dangling bonds on Si NCs can form deep energy levels that enable trapping photogenerated electrons. They proved the existence of dangling bonds by electron paramagnetic resonance signal. For using interface defects, Chung et al. [43] used a defective layer to enhance the synaptic properties of OEAS. Based on conventional indium–gallium–zinc oxide (IGZO) optoelectronic synapse, they introduced an additional quasi-2D IGZO defective layer (2 nm) by spin coating. In the device, the photogenerated carries can be trapped at the defective interface, resulting in a lower de-trapping speed compared with the device without defective layer. In this way, the PPC behavior can be further improved.

2. Heterojunction-induced photogenerated carrier capture

Heterojunction typically involves 2 types of materials. One is the photoactive material that acts as the light absorber and the other acts as the conductive channel. Usually, the photosensitive material can be attached to the conductive channel by spin coating, transfer, deposition, or dripping. Figure 3B shows a typical OEAS with heterojunction [45]. Since the 2 materials have different work functions, the resulting built-in electric field can separate the photogenerated carriers. The photosensitive material can be oxide semiconductors [47,48], perovskite materials [49], 2D materials [50], and sulfur compound [51]. Due to the presence of these materials, the devices tend to have a higher response compared to the VO types. The valence electrons in photosensitive material can be excited to the conduction band after absorbing photons with the increasing of carrier concentration in the conductive channel, resulting in the increase of conductivity. Because of the existence of barrier in built-in electric field, the conductivity will not quickly return to the initial state after removal of light, leading to the exhibition of memory characteristics. This physical phenomenon enables the mimicking of synaptic plasticity. By varying the wavelength, frequency, or intensity of light pulse stimulus, the response intensity of these devices can be effectively controlled.

#### Photo-induced phase transition

The electrical and optical properties of PCMs exhibit a significant contrast on different phases [52–54]. PCMs, based on tellurides and antimonides, exhibit ultrafast electrical switching on the sub-nanosecond time scale [55]. The states can remain highly stable for several years at operating temperatures, which is a critical requirement for nonvolatile memory [56]. Vanadium dioxide (VO_2_), serving as a representative PCM, undergoes a distinctive phase transition from low-temperature monoclinic phase to high-temperature rutile phase at critical temperature of around 341 K [57,58]. During this process, the electrical resistance of VO_2_ undergoes a drastic change by several orders of magnitude.

Li et al. [59] demonstrate an OEAS device by growing VO_2_ thin film on Si wafers. UV light (375 nm) is able to release VO from the VO_2_ lattice because the activation energy to generate VO in VO_2_ is between 3 and 3.5 eV, and the photon energy of 375-nm UV light only satisfies this requirement (3.35 eV) [60]. Meanwhile, the photon energies of red and green light are lower than the activation energy [61,62]. Thus, the device is capable of extracting UV information from the surrounding environment, thereby providing a pathway for neural morphological sensor. The difference in lattice spacing caused by the release of VO and electrons leads to strain in VO_2_, resulting in transition from the low-symmetry monoclinic phase to the high-symmetry rutile phase [63]. Then, the structure of VO_2_ can gradually return to the initial state by applying a negative voltage that can drive oxygen ions back to VO_2_ lattice [64]. This reversible phase transition can be used for optical programming and electrical erasing at room temperature. The response of EPSCs to different pulse numbers are shown in Fig. 3C, where the increasing number of pulse light stimuli promotes the transition from STP to LTP.

#### Interaction of light and ferroelectric materials

Ferroelectric materials have also been used in OEAS devices. The basic principle is based on the interaction of ferroelectric materials and various external factors such as electric fields, stress fields, and optical fields [65–68]. Ferroelectric materials are widely used in nonvolatile memories due to their inherent spontaneous polarization properties. Heterostructures composed of 2D direct-bandgap materials and ferroelectric oxides are considered as potential candidates for emerging semiconductor memory due to their low operating voltage, small size, and excellent nonvolatile properties [69–71].

While ferroelectric polarization reversal is typically achieved by applying an electric field, recent research has increasingly shown that optical-induced methods can also serve as a mean of controlling ferroelectric polarization [72]. As a noncontact method, optical stimulation offers new possibility for the realization of multi-degree-of-freedom devices. Compared to electric field stimulation, optical stimulation can significantly reduce the requirements on circuit design and enhance switching speed [73]. Currently, the application of ferroelectric materials in OEAS mainly exploits the property of optical-induced ferroelectric polarization reversal [74–76].

Figure 3D shows the MoS_2_/BaTiO_3_ (BTO) ferroelectric transistor-based OEAS reported by Du et al [77]. The monolayer MoS_2_ serves as the channel, while the BTO thin film acts as the gate dielectric. The photo-induced polarization state switching after irradiating the device with a triple-pulse light stimulation is illustrated in Fig. 3D. With increasing exposure time, the initially up-polarized region gradually switches to a down-polarized state, while the initially down-polarized region remains unchanged. The photo-controlled ferroelectric polarization switch can be explained by the interaction between photogenerated charges in MoS_2_ and ferroelectric polarization charges in BTO [72]. The conductivity of the device changes under different light stimulation times (wavelength, 450 nm; power density, 10 mW/cm^2^) and shows a good linear relationship with the exposure time.

#### Floating gate effect

The floating gate effect is inspired by the flash memory. The typical structure of this type of OEAS is shown in Fig. 3E including blocking dielectric layer, floating gate, tunneling dielectric layer, and conductive channel layer. The floating gate is usually made of photoactive materials used to achieve the light-stimulated response of OEAS [78]. The tunneling dielectric layer usually has a thin thickness, which is convenient for photogenerated hot carriers to tunnel. Besides, to effectively prevent the recombination of photogenerated carriers, it also needs to have low defects and a large band gap. When stimulated by photons with energy exceeding this bandgap, the floating gate absorbs photons and generates photogenerated carriers, where photogenerated electrons or holes tunnel into the conductive channel, while the other type of carriers are trapped in the floating gate. Thus, the conductivity of the conductive channel is changed, resulting in a photocurrent response. Due to the existence of the dielectric layer, the photogenerated carriers will not recombine immediately after the light stimulus disappears. As a result, the OEAS can have the photocurrent memory characteristics. Wang et al. [79] reported an OEAS with this structure, using CsPbBr_3_ QDs as the floating gate and pentacene as the conductive channel. The device can respond at multiple wavelengths and exhibits good memory properties.

Interestingly, under the same device structure, the floating gate may have different photogenerated electron transfer mechanisms. Zhang et al. [80] introduced an additional gate electrode based on the floating gate structure. They used WSe_2_ as the floating gate material. When a positive bias is applied to the gate, the photogenerated electrons of the WSe_2_ will tunnel to the gate electrode through the gate dielectric. Thus, the holes retained in WSe_2_ will generate an electric field that points from the floating gate to the conductive channel. The electric field will increase the conductivity of the conductive channel, resulting a positive photocurrent. Conversely, when the gate voltage is negative, light stimulation will generate a negative photocurrent.

In the above works, the floating gate serves as the role for light absorption. In addition, the conductive channel can also serve as this role. Sun et al. [81] reported this type of OEAS. They use MoS_2_ as the conductive channel and multilayer graphene as the floating gate material. Under light stimulation, the photogenerated electrons in MoS_2_ tunnel to the graphene, thus forming a negative gate voltage on the floating gate, causing the conductance of the MoS_2_ conductive channel to change.

#### Other types

1. Multi-device integration

OEAS can also be achieved by integrating multiple types of devices and making full use of the functions of each device. For instance, photoresistor and resistive load can be integrated with electrical synapse [78]. Here, the electrical synapse adopts a 3-terminal transistor structure, and the conductive channel is the electrolyte insulator. The photoresistor is connected in series with the resistive load, while the gate of the electrical synapse is connected in parallel with the resistive load. When light stimulates the photoresistor, the resistance of the photoresistor changes. According to Kirchhoff’s law, the voltage on the resistive load changes, which causes the gate voltage of the electrical synapse to change, resulting in the movement of cations in the electrolyte insulator. Therefore, the conductance of the electrical synapse changes, resulting in a current response. When the light stimulation stops, the current response does not disappear immediately due to the slow recovery motion of the metal cations, thus achieving current memory characteristics.

2. Photon-modulated electrochemical doping

Ion migration is also used in this mechanism. Chen et al. [82] reported an OEAS based on an organic 3-terminal electrochemical transistor (OECT). They incorporate a bulk heterojunction as the photoactive layer into the channel of OECTs. An electrolyte is used between the gate electrode and the channel. Light stimulation can perturb the electrochemical doping of channel. When stimulated by light, light-induced charge carriers in the heterojunction will lead to ion transport from the electrolyte to the channel for charge compensation, resulting in electrochemical doping of channel. Thus, the conductance of the channel changes and a photocurrent response is observed. The slow motion of ions enables the memory characteristics of the device.

To summarize OEAS, light as the stimulus signal for OEAS can generally achieve higher bandwidth and lower crosstalk. In the era of AI, the collection and processing of visual information occupies a significant portion of AI tasks. OEAS can be used to simulation the human visual synapses. A network based on OEAS can be used to complete the preprocessing of the optical image at the receiving end, such as direct recognition of simple and feature extraction. In this way, it can reduce the energy consumption of the whole system and improve the processing speed.

At present, the responsivity of OEAS is generally low, especially in the OEASs that rely on defects to achieve memory characteristics. This causes the energy consumption of light stimulation required for OEAS to be still high. Energy consumption is an important evaluation index of synaptic devices. This can be improved by increasing the light absorption. Plasmon effect is an important method. Based on the mechanism of surface plasma resonance, it can effectively enhance the light absorption of the device, thereby increasing the number of photogenerated hot carriers and improving the response of the device [83–85]. In addition, we found that some metal nanostructures can have other unique effects. For instance, OEAS based on Ag nanoparticles can demonstrate both long-term potentiation and long-term depression by light stimulation with different wavelength, which may be related to oxidation and reduction of the Ag nanoparticles under light irradiation [86].

### Optically stimulated optical-response artificial synapses

Here, we call this type of synapses all-optical artificial synapses (OASs). In OAS, both the stimulus and response are optical signals, and synaptic weight modulation is achieved by changing the optical parameters (refractive index or transmittance) of the material or device through optical stimulation and thus affecting the intensity or phase of the optical response signals. Compared with all EAS or OEAS, OASs provide a much faster and energy-efficient way to construct neural network. In addition, light, as an information carrier, has more degrees of freedom compared to electrons, so OASs have lower crosstalk and can be used for multiplexed information processing. Innovative materials such as tungsten trioxide photochromic materials, perovskite photochromic materials, phase-change materials, photochromic materials, and long persistent phosphorescent (LPP) materials have been used in OAS.

**Fig. 4.**
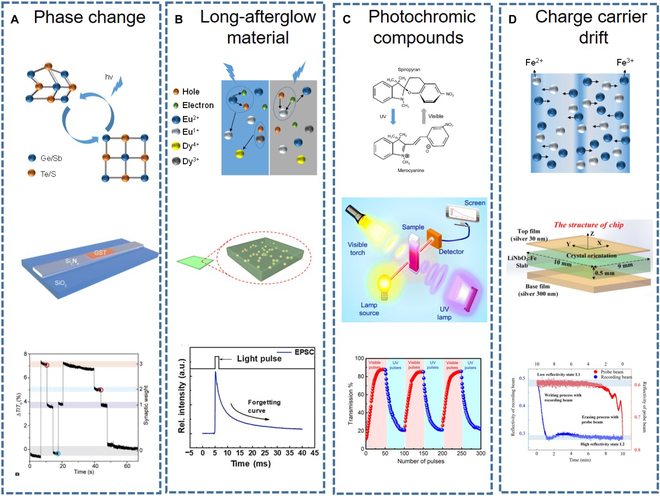
Mechanisms of synaptic plasticity in optically stimulated optical-response artificial synapses. (A) Photo-induced phase transition [87]. (B) Long-afterglow material [9]. (C) Photochromic compounds [88]. (D) Charge carrier drift [89]. Images reprinted with permission from [9,87–89].

PCMs have also been widely used in OAS. Cheng et al. [87] used GST PCMs and integrated silicon nitride waveguides to fabricate OAS, as shown in Fig. 4A. The response mechanism is the transformation of GST material from crystalline to amorphous under light irradiation. Under light stimulation, GST can undergo a transition from the crystalline state to the amorphous state. Different states correspond to different refractive indices, which leads to a change in the coupling between GST and the waveguide, resulting in the change in the transmittance of the waveguide. Thus, the synaptic weight function can be realized. Lu et al. [9] proposed an OAS based on the hybrid of LPP material (SrAl_2_O_4_:Eu^2+^,Dy^3+^) and polydimethylsiloxane (PDMS). As shown in Fig. 4B, the device uses PDMS as a substrate with the LPP material embedded on its surface. The luminescence mechanism of LPP is the formation, transfer, storage, release, and recombination of holes under UV irradiation. This device illustrates typical excitatory postsynaptic intensity (EPSI) behavior of biological synapse, where the output intensity signal reaches a maximum value under UV light stimulation and gradually decays to initial intensity, showing a memory characteristic. Sharma et al. [88] introduced a simple-structure OAS based on the photochromic organic compound called spiropyran. When exposed to UV pulse illumination, the energy of the light pulse can break the C–O bond in the spiropyran molecule, leading to an increase in isomer content and darkening of the color, as shown in Fig. 4C. The increase and decrease in transmittance caused by visible and UV pulses correspond to the EPSI and inhibitory postsynaptic intensity (IPSI) behaviors, respectively. Wei et al. [89] demonstrate an all-photonic synapse device based on a double metal-clad waveguide, as shown in Fig. 4D. The response mechanism is that the internal electric field formed by the drift of electrons and ions under light illumination changes the refractive index of the crystal through nonlinear effects. Therefore, the synapse can be switched in different states with probe and recording light.

In conclusion, OAS possesses the characteristic of light-speed ultralow-energy-consumption computing. It is worth mentioning that light has multiple physical dimensions, such as wavelength, phase, amplitude, and angular momentum, so OAS has the potential to be used for multiplexed computation to achieve high-throughput information processing. Although OASs show advantages in many ways, they still face considerable challenges in terms of integration and manufacturing at scale. Meanwhile, compared to EAS, OASs are still slower in weight modulation speed despite that they have higher computational speeds. Therefore, improving the weight modulation speed and realizing on-chip integration will be an important direction in this field. In addition to being the basic component for all-optical neuromorphic computing, OAS can also be integrated with various optical systems to realize preprocess of optical data. In this way, the energy consumption of these systems can be further reduced. Moreover, OASs are not easily affected by environment, so they have the potential to be used in various extreme environments.

### Electrically stimulated optical-response artificial synapses

This type of artificial synapses can be called EOAS. EOAS has the same function to be used in optical neural networks as OAS because they have also optical response. Compared with light stimulation, electrical stimulation has better programmability and higher precision modulation capabilities [90]. Zheng et al. [91] fabricated a nonvolatile electrically reconfigurable artificial synaptic device with GST phase transitions actuated by heating silicon positive-intrinsic-negative (PIN) diode. The in situ heating has less heat loss compared with the conventional external heater scheme, so the crystallization voltage and amorphous voltage are greatly reduced. At the same time, the silicon PIN structure is compatible with the complementary metal-oxide semiconductor (CMOS) process, which is suitable for the fabrication of large-scale optical field-programmable gate arrays.

### Artificial synapses based on other types of stimulation

Based on the concept of multisensory integration, artificial synapses that can be regulated by other types of stimulus signals such as sound, chemical odor, pressure, and heat have also been extensively studied [92].

#### Artificial synapses stimulated by auditory

Auditory systems are the most efficient and direct means of communication between humans and robots. Therefore, artificial synapses with flexible acoustic sensing capabilities are considered crucial components of human–machine interaction interfaces. Chen et al. [93] reported a synaptic device that achieves STP and LTP functions by modulating the amplitude and pulse duration of an applied surface acoustic wave (SAW). The magnetic film is sandwiched between a pair of interdigitated transducers (IDTs), where the SAW signal is generated by one IDT and the received signal is detected by the other IDTs. The number of skyrmions depends on the amplitude of the SAW. The number of skyrmions increases as the decrease of the SAW sequence interval, which can be regarded as the transition from STP to LTP. Chen et al. [94] studied a novel MXene/MoS_2_-based flexible vibration artificial synapse device. This artificial synapse device, stimulated by sound, achieves high sensitivity for acoustic recognition with a sensitivity of 25.8 mV/dB and a broadband response of 40 to 3,000 Hz. Additionally, they designed and trained a machine learning-based speaker recognition process using an artificial neural network (ANN) with high accuracy (99.1%) for speaker identification.

#### Chemically stimulated artificial synapses

Chemically stimulated artificial synapses are an emerging field in recent years, which can be used to mimic the human olfactory system. Deng et al. [95] proposed a flexible biomimetic olfactory synapse based on a porous solid polymer electrolyte (SPE). The device has excellent sensitivity to hydrogen sulfide (H_2_S) and memory capability for cumulative effects. When the device is in an H_2_S atmosphere, the SPE can establish ion association with H_2_S, electrostatic interaction between weak cations and anions, forming and enhancing ion migration. In addition, after the gate pulse is removed, the doped cations in the polymer poly (3,4-ethylenedi-oxythiophene) doped with poly (styrene sulfonate) (PEDOT:PSS) channel cannot immediately diffuse back into the SPE, showing relaxation behavior. The release of H_2_S was detected to evaluate rotten eggs, and the conversion from short-term memory (STM) to long-term memory (LTM) was successfully demonstrated by adjusting the pulse duration. Hu et al. [96] demonstrated an artificial olfactory chemical-resistant synapse composed of 3D layered WO_3_@WO_3_ nanofibers. These nanofibers exhibit a persistent electrical resistance response due to the strong adsorption of water molecules. By using recurrent neural networks to process time-dependent data, they successfully identified gaseous chemical substances such as 3-hydroxy-2-butanone, triethylamine, and trimethylamine. This research achieved gas recognition with an accuracy of over 90% without the need for training. It provides a new avenue for artificial olfactory system research and holds vast potential for applications.

#### Pressure-stimulated artificial synapses

The development of low-power, low-cost, low-complexity, and high-efficiency artificial synapse tactile sensing systems holds tremendous potential in Internet of Things (IoT) and AI. Jia et al. [97] demonstrate a mechanoplastic semi-floating gate transistor artificial synapse based on integrated graphene/h-BN/tungsten diselenide heterostructures and triboelectric nanogenerators (TENGs). The operating mechanism is attributed to carrier trapping and detrapping in the graphene layer. This device exhibits synaptic plasticity with an ultra-low energy consumption of 74.2 fJ per synaptic event. Zhang et al. [98] developed a self-powered tactile sensing system, which is expected to advance the field of intelligent perception . The gas-ions-gated (GIG) transistor serves as the artificial synapse, while the triboelectric plasma acts both as the tactile sensor and as the driver signal for the GIG transistor. Triboelectric plasma contains N^2+^ ions that directly adhere to the graphene surface, functioning as a floating gate to modulate its electrical transport properties.

#### Temperature-stimulated artificial synapses

Temperature-sensitive artificial synapse devices play a crucial role in future electronic skin and intelligent sensing systems. Based on temperature stimulation, artificial synapses can achieve rapid and accurate responses to temperature changes, creating a more intelligent and sensitive perception environment for humans. Han et al. [99] demonstrated an artificial synapse for multimodal perception that simultaneously receives visual and thermal stimuli and converts them into electrical signals. This unique pattern is used for fingerprint identification, where the fingerprint pattern is identified by light and real skin or fake skin is identified by heat. Duan et al. [100] proposed a bioinspired tactile-temperature fusion artificial synapse device based on the volatility of VO_2_. It utilizes the inherent thermal sensitivity of the metal-insulator transition in VO_2_ to sense temperature. This device can be used to integrate multiple spatially related sensory stimuli, such as touch and temperature, to identify Braille characters. It provides new insights into neuromorphic perception and neuromorphic computing based on sensory integration.

#### Magnetically stimulated artificial synapses

The artificial synapse based on magnetic stimulation achieves switching between HRS and LRS through the magnetic-electric (ME) coupling mechanism [101]. The magnetoresistive element converts magnetic signals into induced voltages, where the ME voltage coefficient is considered as the synaptic weight. The ferroelectric tunnel junction (FTJ) is one of the simplest structures for magnetic stimulation artificial synapses. Electrons tunnel through an ultra-thin ferroelectric barrier, relying on the polarization of the ferroelectric material. Lu et al. [102] presented a novel artificial synapse device based on Cu/P(VDF-TrFE)/Ni. This device exhibits significant nonlinear magnetoelectric effects and flexible nonvolatile multi-level memory at room temperature. Binary information is encoded by changing the magnetoelectric voltage coefficient (αE) of the magnetoresistive element through selective electric field pulses. Under pulsed voltage, the state of αE can repetitively switch among 2*n* states (*n* =1, 2, 3) in a zero DC bias magnetic field. αE is used as the weight of the synapse, while the induced magnetoelectric voltage V_ME_ is considered the postsynaptic potential (excitatory or inhibitory). Shen et al. [103] demonstrated the switchable behavior of a multiferroic heterostructure memory resistor, which comprises a piezoelectric layer [Pb_0.7_(Mg_1/3_Nb_2/3_) O_3-0.3_PbTiO_3_ or PMN-PT] and 2 magnetostrictive layers (Ni). The ME coupling mechanism in this heterostructure is achieved through the interface transfer of strain induced by either the piezoelectric behavior of PMN-PT or the magnetostriction of Ni. Jia et al. [97] developed a perception system comprising a remote tactile tip and magnetic synapses. The system exhibits high sensitivity and a wide dynamic range and can detect pressures as low as 6 Pa. With separate touch and sensing components, it can measure tactile stimuli up to 1,000 Hz without distortion. Its excellent performance in surface texture recognition, wrist pulse measurement, and underwater detection highlight its significant potential for various mechanical sensing applications in different environments.

## Applications of Artificial Synapse Devices

The development of artificial synapses has promoted the advancement of in-sensor and in-memory computing. With the diversification of device functions and the maturity of fabrication technology, this field has gradually developed from the early demonstration of synaptic plasticity behavior to the realization of complex AI applications.

### Vision tasks

Vision is the main means for humans to obtain information. Therefore, current neuromorphic applications are mainly centered around the perception and processing of visual information.

#### Image perception

Image perception is the most basic function of the biological visual nervous system. Currently, many studies have also achieved this function through OEAS. Zhu et al. [49] demonstrated image reinforcement learning based on PPF of OEAS devices. By increasing the number of light pulses and the optical power density, the on-current steadily increases. A larger on-current represents higher-quality image sensing, akin to how the human brain forms deeper impressions through learning and training. This process demonstrates the function of neuromorphic pattern reinforcement. Additionally, synaptic plasticity includes the characteristic of relearning. Gao et al. [48] demonstrated this function in OEAS. That is, it usually takes less time to relearn previously memorized but lost information. Based on this, Li et al. [45] used CsPbBr_3_/graphene OEAS to demonstrate the process of the human brain perceiving and learning images, which is the typical “learning–forgetting–relearning–forgetting again” cycle. In perceptual learning, the learning process distinguishes useful input signals from a large amount of information and transforms them into effective recognition signals. With the accumulation of learning experience, the ability to differentiate information gradually improves. The realization of human perceptual learning has great potential for high-performance AI perception devices.

#### Image preprocessing

**Fig. 5.**
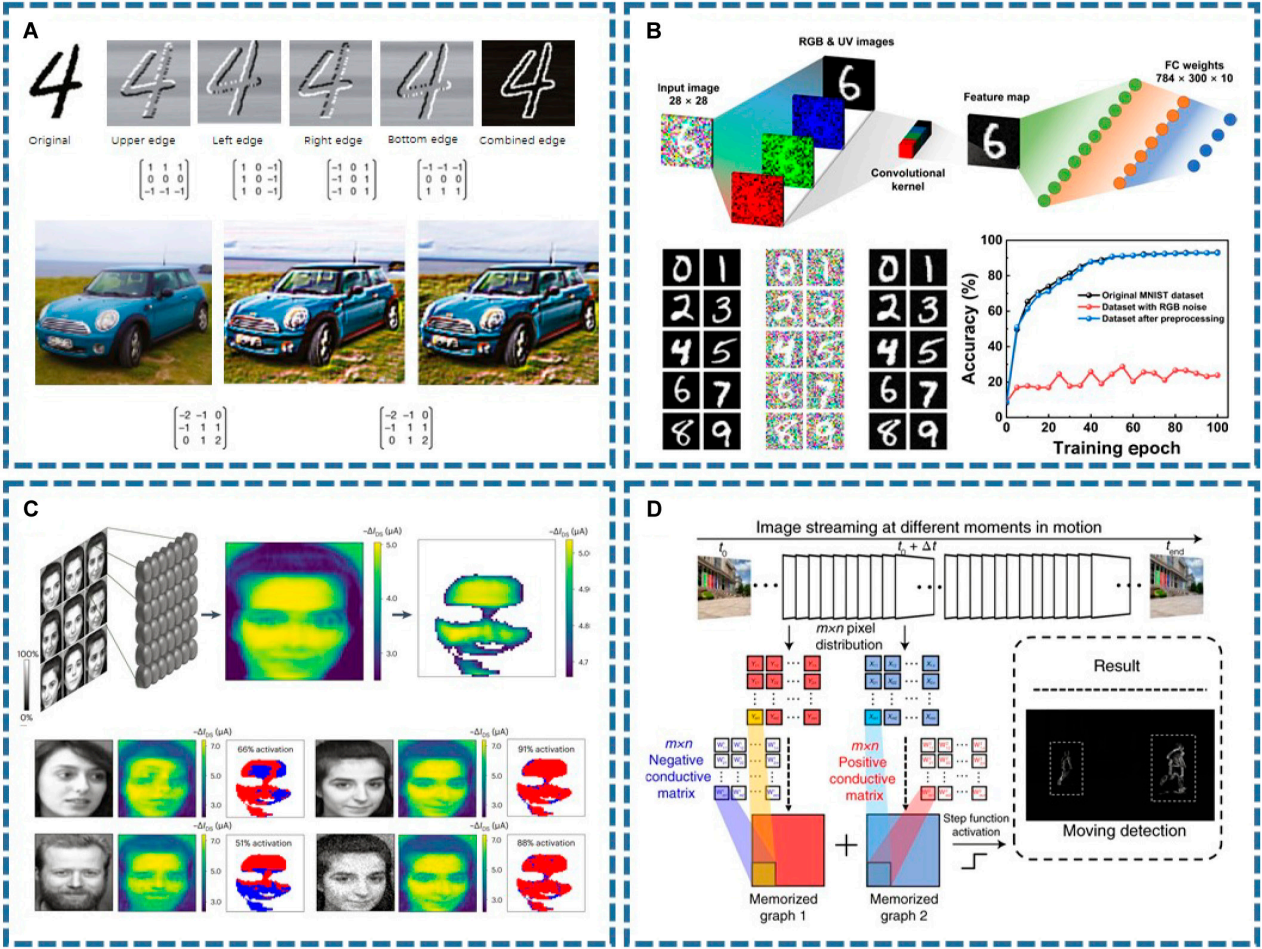
Visual tasks based on artificial synapses. (A) Convolution kernel performs image edge detection [106]. (B) Image preprocessing and recognition [59]. (C) Face recognition [82]. (D) Gesture recognition [80]. Images reprinted with permission from [59,80,82,106].

Image preprocessing is used for effective information extraction or image downscale, thereby reducing the amount of computation required for further processing by the subsequent network. In this way, computation speed can be accelerated and the energy consumption can be reduced. ANNs, based on OEAS or OAS, can realize preprocessing of analog optical images. First, the long-term memory characteristics of OEAS can be used to enhance the contrast of perceived images [104]. When a weak light signal is used to repeatedly stimulate the OEAS array, the device illuminated by the effective signal will produce a stronger light response and have a longer current memory time compared with the device illuminated by the noise signals. After a period of forgetting, the difference in photocurrent between the 2 increases, indicating an enhancement in image contrast. In addition, OEAS and OAS can realize image edge detection. Wang et al. [105] demonstrate a dual-modal OEAS based on the hybrid structure of Si NCs and poly(3-hexylthiophene) (P3HT), which is capable of working in 2-terminal and 3-terminal modes. In the 2-terminal mode, they demonstrated the edge detection of images in simulations. At different threshold frequency, the device can realize detection of different image details. Feldmann et al. [106] reported an OAS array based on PCM integrated optical waveguides, which can realize the function of convolution kernel, as shown in Fig. 5A. By encoding different weights in the devices, the convolution kernel can perform different image edge detection tasks. Subsequently, based on the PCM integrated optical waveguides. Wen Zhou et al. [90] reported an artificial synapse array with electrical stimulation and light response. Compared with OAS, electrical stimulation has higher programming flexibility and scalability. Edge detection was also demonstrated by their chip.

#### Image recognition

At present, the processing of visual information in the visible band has received widespread attention. In addition to the visible wavelengths, the invisible light also contains many important information in animal and plant behavior. Li et al. [59] extracted UV light information from the environment by using the different responses of VO_2_ artificial synapse devices in UV light and visible light. This information extraction behavior, which focuses only on specific colors, performs a weighted averaging operation on pixels in a small area through an appropriate convolutional control. A UV vision system with preprocessing and recognition functions is shown in Fig. 5B. The image recognition accuracies of 3 types of test datasets [original grayscale Mixed National Institute of Standards and Technology (MNIST) test dataset, the MNIST test datasets adding RGB Gaussian noise, and the weighted average MNIST test datasets processed by the sensor] show great differences. This result shows the effectiveness of the device in extracting UV information. Chen et al. [82] report a photonic organic synapse based on photon-modulated electrochemical doping. A layer of synapse array was demonstrated acting as an artificial retina, as shown in Fig. 5C. When the facial images are input in the form of light intensity, the array can sense the light information and reconstruct the images showing the key information of the face. The information can then be used for face recognition. Seo et al. [107] simulated the color and color-mixed pattern recognition of the human visual system using artificial synapses and optical sensing functions based on h-BN/WSe_2_ heterojunctions. The OEAS exhibits nearly linear weight updates, with the weight change for each conductance state being less than 1%. The OEAS device can operate at a read voltage of 0.3 V, with each spike consuming only 66 fJ. It enables accurate and energy-efficient color and color-mixed pattern recognition, suitable for more complex pattern recognition tasks.

#### Motion detection and recognition

A core requirement for various intelligent scenarios such as artificial vision, security monitoring, driverless cars, and military defense is motion detection and recognition (MDR). Zhang et al. [80] constructed a 2D retinomorphic artificial synaptic device that integrates optical perception, memory, and computation to implement MDR. Pulsed light can induce positive photocurrents and negative photocurrents at different gate voltages, which is similar to how human retinal bipolar cells divide visual signals into ON and OFF signals. By applying different gate voltages to the devices in an array to enable the array to function as a convolutional kernel, moving objects in the video can be detected and extracted (Fig. 5D). Recently, Zhou et al. [108] reported an event-driven vision sensor unit by using bidirectional floating gate transistors with a parallel structure of capacitor and resistor. This vision unit only generates photocurrent pulses in response to changes in light intensity. Therefore, it can realize the function of event camera, that is, extract only moving objects in visual information. Besides, the structure of the floating gate enables the intensity encoding of photocurrent, thereby realizing the in-sensor recognition of moving objects.

### Nonvisual tasks

Using artificial synapses to simulate the synaptic plasticity and neural processing capabilities in the human brain, artificial synapses also have significant applications in many nonvisual fields.

#### Edge learning

**Fig. 6.**
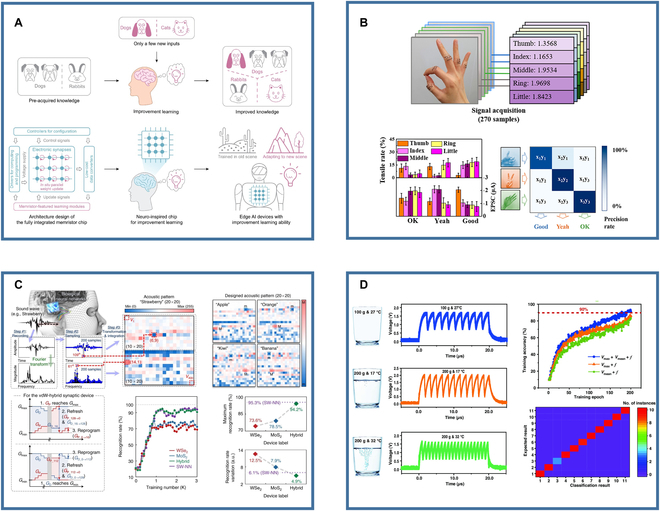
Nonvisual tasks based on artificial synapses. (A) Edge learning [109]. (B) Motion detection and recognition [110]. (C) Voice recognition [111]. (D) Braille recognition [100]. Images reprinted with permission from [100,109–111].

Edge learning is an important application in neuromorphic computing. Edge learning is an energy-intensive training, which requires low energy consumption and high training speed. The in-memory computing configuration of artificial synapses effectively circumvents the substantial energy consumption resulting from extensive data movement. Zhang et al. [109] developed a fully integrated memristor crossbar array memory chip for executing edge learning tasks, such as motion control, image classification, and speech recognition as shown in Fig. 6A. This chip demonstrated advantages in data processing speed, energy efficiency, and real-time environmental adaptability when performing edge learning tasks such as motion control, image classification, and speech recognition. This research is poised to drive the development of edge computing and intelligent systems, providing critical support for future smart devices and IoT technologies.

#### Multimodal signal recognition/processing

Artificial synapses stimulated by different modalities can be used to process or recognize corresponding modal information. In tactile tasks, artificial synapses can perceive and respond to touch, enhancing the flexibility and interaction capabilities of robots and prosthetic devices. Voice-stimulated artificial synapses can be used for acoustic signal processing, which may have potential application in hearing aids, cochlear implants, and speech-activated interfaces. Additionally, in olfactory tasks, chemically stimulated artificial synapses have been used to detect and differentiate various compounds, playing an important role in environmental monitoring, food safety, and quality control.

Liu et al. [110] fabricated a stretchable nanowire neuromorphic transistor artificial synapse that can sense tactile information and perform gesture recognition, as shown in Fig. 6B. The device can sense skin deformations under close contact. Different degrees of deformation can stimulate the device to produce different response signals. The authors pasted 5 devices on each of the 5 fingers of one hand. The 5 devices respond differently to different gestures, after which these responses are processed by an ANN to realize gesture recognition.

Seo et al. [111] used van der Waals hybrid synaptic devices for acoustic pattern recognition, as shown in Fig. 6C. They first converted sound signals into acoustic patterns and prepared a dataset, validating the device’s feasibility in hardware neural networks (HW-NNs). Sound signals were transformed into time or frequency functions, sampled to generate 20 × 20 pixels acoustic images of 5 words for training and inference. They designed a single-layer ANN and applied the sigmoid activation function to convert acoustic pattern pixels into the input neuron layer, updating synaptic weights via the backpropagation algorithm. This resulted in establishing an HW-NN capable of acoustic and MNIST digit pattern recognition. Chen et al. [94] reported a sound recognition system based on MXene/MoS_2_ flexible vibration artificial synapses. Due to its excellent sound recording properties, MXene/MoS_2_ flexible vibration sensor (FVS) shows enormous potential as a voice recognition device. It was used to record piano notes (“Do,” “Re,” “Mi,” “Fa,” and “So”). The voltage variations when stimulated by different note intensities and at the same intensity matched well, demonstrating excellent recording accuracy and sensitivity.

Duan et al. [100] utilized artificial synapses to detect and encode pressure and temperature to recognize braille, as shown in Fig. 6D. These synapses integrate tactile and temperature information and exhibit cross-modal perception capabilities. They constructed a multilayer perceptron network to recognize multimodal environmental events (pressure and temperature), highlighting their potential and application prospects in multisensory fusion.

## Conclusion and Outlook

**Fig. 7.**
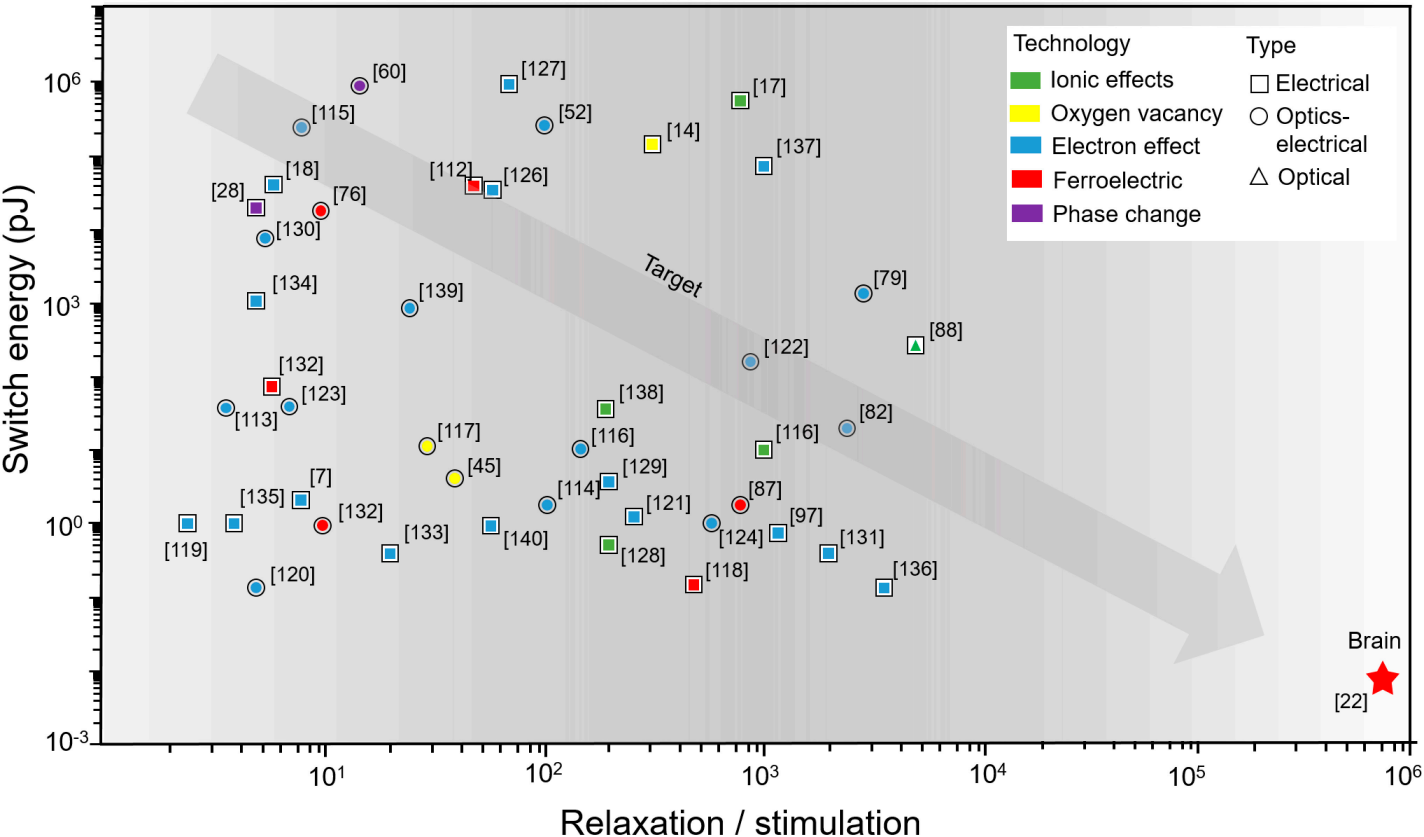
The performance indicators of artificial synapses with various response mechanisms.

We have summarized the performance of electrical, optoelectronic, and OAS, as shown in Fig. 7 [112–140]. As one of the key indicators, the *x* axis shows the ratio of the post-response recovery time to stimulus time. This ratio is often used to describe the dynamic response characteristics of artificial synapses. Optimizing this ratio allows the artificial synapse to better perform perceptual learning and mimic the human memory function. The *y* axis is the energy consumption of each synaptic event. Reducing energy consumption is a common goal pursued by all the artificial synapses. Unfortunately, we are still a long way from the human brain (1 fJ/synaptic event [22]) in this direction.

Among them, the principle of electron effect has been the most widely used. Artificial synapses based on PCMs have the highest conversion energy. Compared with optical stimulation, EAS have lower weight modulation power consumption. However, OASs have lower computational power consumption. OAS has a higher post-response recovery time, which means that it is more suitable for artificial synapses in memory and recognition tasks. For neuromorphic computing, we still do not know which strategy is better, optical, optoelectronic, or all-electric. This will take time to prove. For now, they each have their own advantages and limitations, which we have discussed in each chapter. The large-scale integration of nanoscale artificial synapses faces common challenges such as thermal diffusion, cross-talk, and circuit design issues. Achieving power consumption levels comparable to or approaching those of biological synapses in individual artificial synapse devices is a challenge that remains to be addressed. In addition, realizing artificial synapse devices with improved responsiveness and the ability to detect small signals comparable to biological synapses is also a goal that needs to be achieved.

The research and development of multimodal artificial synapses with biological synaptic functions signify significant progress in the field of AI. To mimic the human brain’s nervous system, existing multimodal synapses should be combined and developed into an organic whole. In such a multimodal neuromorphic system, optical-stimulated synapses mimic visual sensing, odor-stimulated synapses mimic olfactory sensing, pressure-stimulated synapses mimic tactile sensing, sound-stimulated synapses mimic auditory sensing, and temperature-stimulated synapses mimic temperature sensing. These synapses perform the first step of preprocessing the external information and then pass the signals to the all-electrical synapse network for the final AI task. A great application scenario for this system would be edge computing on robots to realize a real anthropomorphic intelligent robots. OAS, as a special case, can be used to build all-optical neural network processing of optical signals. For other types of signals, they need to be converted to optical signals by active optoelectronic devices [141].
